# Acknowledgement of Reviewers

**DOI:** 10.1111/jdi.14106

**Published:** 2023-11-30

The publication of invaluable papers in the *Journal of Diabetes Investigation* depends on the prompt, careful review of submitted manuscripts. We would like to thank the Editorial Board members, the International Editorial Board members, the Assistant Editorial Board members and the following experts for reviewing manuscripts from September 1, 2022 to August 31, 2023.

Juthi Adhikari

Vasudha Ahuja

Toru Aizawa

Satoru Akazawa

Gulali Aktas

Nurshad Ali

Reza Alizadeh‐Navaei

Ziyad Saeed Almalki

Hidenori Arai

Atsushi Araki

Shin‐ichi Araki

Noriko Asahara

Shunichiro Asahara

Kenji Ashida

Yoshimasa Aso

Motoharu Awazawa

Ahmad Khusairi Azemi

Masayuki Baba

Tetsuya Babazono

Megu Yamaguchi‐Baden

J Baelde

Yasuko K Bando

Pilar Isabel Beato‐Víbora

Henning Beck‐Nielsen

Shahbanoo Bidari

Ryotaro Bouchi

Alex Brito

Shaohang Cai

Wenfu Cao

Te‐Fu Chan

Shin‐Tsu Chang

Sanjay Chatterjee

Fengling Chen

Harn‐Shen Chen

Huiling Chen

Liangmei Chen

Ming‐Wei Chen

Szu‐Chi Chen

Kyu Yong Cho

Mi‐Kyoung Cho

Eun Jung Choi

Soo Choi

Sung Hee Choi

Won‐Il Choi

Daisuke Chujo

Dana Mihaela Ciobanu

Ohad Cohen

Xiaona Cui

Alessandra Da Silva

Makoto Daimon

Undurti Das

Takahisa Deguchi

Heyuan Ding

Kentaro Doi

Zheyi Dong

Tuba Taslamacioglu Duman

Jun Eguchi

Masanori Emoto

Ihsan Esen

Toshihito Fujii

Rumi Fujikawa

Junji Fujikura

Shimpei Fujimoto

Shiho Fujisaka

Tomomi Fujisawa

Yoshihito Fujita

Yukihiro Fujita

Yoshio Fujitani

Kei Fukami

Maki Fukami

Michiaki Fukui

Tomoyasu Fukui

Shinya Furukawa

Hiroto Furuta

Siti Maisharah Sheikh Ghadzi

Atsushi Goto

Yunjuan Gu

Yashdeep Gupta

Kyoung Hwa Ha

Yuriko Hajika

Masahide Hamaguchi

Akihiro Hamasaki

Ko Hanai

Masumi Hara

Ai Harashima

Koshi Hashimoto

Yoshitaka Hashimoto

Yuji Hataya

Akinori Hayashi

Yoshitaka Hayashi

Yasuaki Hayashino

Mayu Higashioka

Tatsuhito Himeno

Tsutomu Hirano

Takahisa Hirose

Inoue Hiroshi

Yushi Hirota

Ichiro Horie

Yukio Horikawa

Masayuki Hosoi

Tetsuya Hosooka

Kaijian Hou

Chang‐Hsun Hsieh

Song Hualin

CN Huang

Yasuo Ido

Noriko Ihana‐Sugiyama

Katsumi Iizuka

Kaori Ikeda

Takayuki Ikeda

Fumiaki Imamura

Minako Imamura

Takeshi Inagaki

Reiko Inagi

Marco Infante

Toyoshi Inoguchi

Junichiro Irie

Shun Ishibashi

Yasushi Ishigaki

Hisamitsu Ishihara

Mohammad Safiqul Islam

Yoshiya Ito

Masato Iwabu

Naoko Iwasaki

Minoru Iwata

Jae‐Han Jeon

Ying Jiang

Sang‐Man Jin

Ji Eun Jun

Hye Seung Jung

N. K. Elnaggar

Tomoyuki Kabutoya

Nozomu Kamei

Naotetsu Kanamoto

Akio Kanazawa

Yoshinori Kanemaru

Hideaki Kaneto

Hyeon‐Ji Kang

Soji Kasayama

Atsunori Kashiwagi

Hirokazu Kashiwagi

Naoto Katakami

Hisaya Kato

Koichi Kato

Takehiro Kato

Yoshiro Kato

Takumi Kawaguchi

Dan Kawamori

Ryoichi Kawamura

Tomoyuki Kawamura

Daiji Kawanami

Eiji Kawasaki

Mahesh Kumar Khanal

Altaisaikhan Khasag

Touch Khun

Takeshi Kikuchi

Touru Kikuchi

Chong Hwa Kim

Doo‐Man Kim

Tomohiko Kimura

Tomonori Kimura

Shunbun Kita

Munehiro Kitada

Tadahiro Kitamura

Atsushi Kiyota

Junji Kobayashi

Noriko Kodani

Kateryna Kolesnikova

Mitsuhisa Komatsu

Tatsuya Kondo

Masaya Koshizaka

Junji Kozawa

Garry Kuan

Teru Kumagi

Shinji Kume

Akio Kuroda

Yoshiki Kusunoki

Hitoshi Kuwata

Hyuk‐Sang Kwon

Davide Lauro

Chi‐Ho Lee

Christopher Lee

Eun Young Lee

I‐Te Lee

Sung Eun Lee

Ting‐Wei Lee

Won‐Young Lee

Yongho Lee

Quanmin Li

Sheyu Li

Xinyu Li

Biaoyang Lin

Chia‐Hung Lin

Liang‐Yu Lin

Shin‐Yu Lin

Weiyin Vivian Liu

Yujie Liu

Chieh‐Hua Lu

Fei Luo

Sihui Luo

Shuquan Lv

Shiro Maeda

Yoshiro Maezawa

Rayaz Malik

Dasaku Masuda

Masafumi Matsuda

Yuki Matsuhashi

Munehide Matsuhisa

Yoshihisa Matsukawa

Takeshi Matsumura

Takashi Matsuoka

Mohd Zulfakar Mazlan

Shu Meguro

Misganaw Mengstie

Hirohito Metoki

Takashi Miida

Takashi Miki

Takayuki Miki

Akira Mima

Isao Minami

Tomoya Mita

Akio Mitani

Junnosuke Miura

Teruki Miyake

Satoshi Miyamoto

Hideaki Miyoshi

Toru Miyoshi

Hiroki Mizukami

Mie Mochizuki

Himel Mondal

Joon Ho Moon

Jesus Moreno‐Fernandez

Katsutaro Morino

Tomoaki Morioka

Yoshiaki Morishita

Yoshiyuki Morishita

Shota Moyama

Liangshan Mu

Seiichi Munesue

Chieko Murakami

Kazutoshi Murakami

Takaaki Murakami

Naoya Murao

Mototsugu Nagao

Shoichiro Nagasaka

Hisamichi Naito

Miki Nakajima

Akinobu Nakamura

Shuhei Nakanishi

Eitaro Nakashima

Naoki Nakashima

Yusuke Nakatsu

Atsuko Nakatsuka

Takao Nammo

Akihiko Narisada

Chrinetine Newman

Qin Xiang Ng

Akihiko Nishikimi

Rimei Nishimura

Yuichi Nishioka

Jinliang Niu

Mitsuhiko Noda

Takashi Nomiyama

Hiroshi Nomoto

Masatoshi Nomura

Shinsuke Noso

Makiko Ogata

Sumito Ogawa

Masahito Ogura

Shoko Ohno

Mitsuru Ohsugi

Yasuharu Ohta

Yoichi Oikawa

Akira Okada

Yosuke Okada

Takayuki Okamoto

David ONeal

Yukiko Onishi

Atsuhiko Ota

Qi Pan

Jeong Hyun Park

Jiyun Park

Peramaiyan Rajendran

Venkitachalam Ramanarayanan

Xingwu Ran

Sang Youl Rhee

Jenna L. Riis

Grace Russell

Yoshifumi Saisho

Yoshihiko Saito

Kazuhiko Sakaguchi

Mashito Sakai

Hiroshi Sakaue

Iman I. Salama

Noemi Salmeri

Kazunori Sango

Motoaki Sano

Abdur Razzaque Sarker

Shugo Sasaki

Takayoshi Sasako

Toshiyasu Sasaoka

Asako Sato

Junko Sato

Yunfeng Shen

Kenichi Shikata

Michio Shimabukuro

Akira Shimada

Kenju Shimomura

Dai Shimono

Jun Shirakawa

Khalid Siddiqui

Muhammad Siddiqui

Masakatsu Sone

Swayam Srivastava

Paweł Stanirowski

Jian‐bin Su

Ming Su

Chakrabarti Subrata

Kazuhiro Sugimoto

Ken Sugimoto

Yoshihisa Sugimura

Takehiro Sugiyama

Zilin Sun

Hirotsugu Suwanai

Atushi Suzuki

Junichi Suzuki

Ryo Suzuki

Shigeru Suzuki

Kiyoshi Suzuma

Yasuharu Tabara

Hayato Tada

Yuji Tajiri

Mitsuyoshi Takahara

Hirokazu Takahashi

Kazuma Takahashi

Yoshihiro Takahashi

Ken Takao

Takeshi Takayanagi

Fumihiko Takeuchi

Yumi Takiyama

Yoshikazu Tamori

Yoshifumi Tamura

Chia‐Hui Tan

Katsuya Tanabe

Daisuke Tanaka

Nagaaki Tanaka

Hirotaka Tashiro

Mitra Tavakoli

Yasuo Terauchi

Keiichi Torimoto

Masao Toyoda

Kotaro Tsuboi

Kyoichiro Tsuchiya

Satoru Tsujii

Tao‐Hsin Tung

Yohei Ueda

Shinji Ueno

Satoshi Ugi

Hiroyuki Unoki‐Kubota

Emi Ushigome

Isao Usui

Johan Verhaeghe

Chaofan Wang

Chih Yuan Wang

Haining Wang

Jun‐Sing Wang

Qiuyue Wang

XinLei Wang

Kentaro Watanabe

Jong Chul Won

Chung‐Ze Wu

Tao Wu

Zekai Wu

Weizhen Xi

Kunimasa Yagi

Hiroaki Yagyu

Yuichiro Yamada

Sho‐ichi Yamagishi

Kousuke Yamahara

Yasuhiko Yamamoto

Shunsuke Yamane

Tomoya Yamashita

Katsuya Yamazaki

Yuji Yamazaki

Yuan‐Horng Yan

Kun Yang

Tao Yang

Zahra Yari

Hiroshi Yatsuya

Hiroshi Yokomichi

Hisashi Yokomizo

Takashi Yoneda

Hideto Yonekura

Tohru Yorifuji

Satoshi Yoshiji

Xuefeng Yu

Yongfu Yu

Gang Yuan

Fujita Yukihiro

Seyed Mohsen Aghaei Zarch

Mingliang Zhang

Wei Zhang

Xiaomin Zhang

Yanling Zhang

Haoming Zhou.

